# Using Attachment and Biobehavioral Catch-up with young children with developmental delays: A multiple-baseline trial of attachment, sensitivity, and cortisol

**DOI:** 10.1177/17446295221104614

**Published:** 2022-06-01

**Authors:** Ahmed Riaz Mohamed, Paula Sterkenburg, Joshua G. Yeatman, Esmé van Rensburg, Carlo Schuengel

**Affiliations:** Department of Clinical Child and Family Studies and Amsterdam Public Health Research Institute, 1190 Vrije Universiteit, Amsterdam, Netherlands; Department of Psychology, 56405 North-West University, Potchefstroom, South Africa; Department of Psychology, 56410 University of Pretoria, Pretoria, South Africa; Department of Clinical Child and Family Studies and Amsterdam Public Health Research Institute, 1190 Vrije Universiteit, Amsterdam, Netherlands; Department of Assessment and Treatment, 61165 Bartiméus, Doorn, Netherlands; Department of Psychology, 56410 Universityof Pretoria, Pretoria, South Africa; Department of Psychology, 56405 North-West University, Potchefstroom, South Africa; Department of Clinical Child and Family Studies and Amsterdam Public Health Research Institute, 1190 Vrije Universiteit, Amsterdam, Netherlands

**Keywords:** attachment and biobehavioral catchup, attachment-based intervention, developmental delays, intellectual disabilities, multiple-baseline single-case design

## Abstract

The Attachment and Biobehavioral Catchup intervention potentially offsets psychosocial risks facing dyads in which children have intellectual disability or developmental delays. In this single-case multiple-baseline study the efficacy of this intervention was tested across three such South African families. Maternal sensitivity, attachment security, and child affect regulation were measured weekly during a baseline and intervention period, using the Ainsworth Maternal Sensitivity Scales, Attachment Q-sort and salivary cortisol, respectively. Furthermore, post-intervention interviews invited parents’ and intervenors’ evaluations of the intervention. Visual analysis broadly indicated improvement in maternal sensitivity and attachment security across subjects over time following the introduction of the intervention, although randomisation tests were not statistically significant. Effects on affect regulation were not clearly observed and may have been influenced by case-specific variables. Parent-participants and intervenors also identified particularly helpful contributions from the intervention. Findings underscore the importance of individual-level effects evaluation, especially when implementing interventions outside the original population.

The prevalence of intellectual disabilities in developing contexts such as Asia and Africa exceeds that in the developed world (Olusanya et al., 2020). Yet, publicly-funded screening, assessment and care for people with intellectual disabilities in South Africa is limited and specific training for health professionals remains inadequate (McKenzie et al., 2013). There is a need, therefore, for accessible, efficacious and sustainable interventions targeted at the mental wellbeing of people with intellectual disabilities that do not further burden already stretched public resources. Attachment-based interventions have been advanced as important avenues for time-limited and resource-light interventions for children’s difficulties (Bakermans-Kranenburg et al., 2005). This is particularly relevant given the links that have been identified between attachment problems and poorer social competence as well as behavioural and emotional challenges across the developmental spectrum (Groh et al., 2012, 2017). This is of concern when considering that children with intellectual disabilities may be at greater risk of developing insecure attachment relationships (Feniger-Schaal and Joels, 2018; Ganiban et al., 2000). Janssen et al.’s (2002) stress-attachment model posits that this may place them at an elevated risk of poorer mental health given the role of problematic attachment in the development of affect dysregulation and its relation to the development of psychopathology. Following from this, the present study sought to explore the efficacy of the Attachment and Biobehavioral Catchup (ABC; Dozier and Bernard, 2019) intervention in three South African dyads in which the child had an intellectual disability or developmental delays.

Sensitive responsiveness may be particularly impeded in parents of children with intellectual disabilities due to the anomalous and often idiosyncratic signal repertoire of these children and syndrome-specific manifestations of affect regulation and social information processing (Atkinson et al., 1999; Cicchetti and Serafica, 1981; Feniger-Schaal et al., 2019). This may explain the higher risk of attachment insecurity in children with intellectual disabilities and emphasises the importance of such interventions for this population. Meta-analytic evidence has shown that relatively brief interventions that target caregiver sensitivity have been particularly impactful in enhancing attachment security in children (Bakermans-Kranenburg et al., 2003). Such interventions may, therefore, serve to remediate the underdeveloped affect regulatory processes embedded within less secure caregiver-child relationships (Schuengel et al., 2009) by enhancing the caregiver’s capacities as both a secure base and safe haven for the child. However, despite the clinically-relevant potential of attachment-based interventions, there has been only limited investigation in this area for children with intellectual disabilities and developmental delays (see Mohamed and Mkabile, 2015; Platje et al., 2018; Sterkenburg et al., 2008b).

The ABC intervention is an attachment-based parenting programme carried out over 10 home visits. The programme was originally designed for the caregivers of infants and young children at risk of developing disorganised attachments such as those who have been maltreated or neglected. Drawing on attachment theory and stress neurobiology, its targets are: to foster improved nurturing behaviours towards the child when distressed, to reduce intrusive and frightening parenting behaviours, and to increase affective synchrony at times when the child is not distressed. These targets are intended to facilitate caregiver sensitivity and—by extension—child attachment security and affect regulation (Dozier and Bernard, 2017, 2019). Each session is guided by a theme that aligns directly with the intervention targets and includes in-vivo commenting and video-feedback to reinforce positive, and reduce negative, parent-child interactions. In-session learning is augmented with applied homework exercises which are discussed during subsequent sessions. Strengths and existing parenting capacities take the foreground in ABC, rather than shortfalls. Multiple randomised controlled trials have demonstrated the role of the ABC in improving caregiver sensitivity (Berlin et al., 2018; Hepworth et al., 2020; Yarger et al., 2020), attachment security (Bernard et al., 2012; Zajac et al., 2020), as well as cortisol regulation (Bernard et al., 2015; Dozier et al., 2008; Garnett et al., 2020) among those who received the ABC versus a control intervention.

The ABC has to date, however, not been tested in the context of children with intellectual disabilities or developmental delays, whose parents may especially seek in-the-moment feedback to encode their child’s anomalous signals (Janssen et al., 2002). This study aimed to assist with understanding how the intervention may work in these dyads and how clinically relevant change may manifest itself. In doing so, the outcomes in the current study—caregiver sensitivity, attachment security and cortisol regulation—were continuously assessed, over time, during a baseline and intervention period. The following hypotheses were tested:

1. Caregiver sensitivity will show an increase over time, and across dyads, following the introduction of the ABC intervention.
2. Attachment security will show an increase over time, and across dyads, following the introduction of the ABC intervention.
3. Hypercorticolism (persistently elevated diurnal cortisol levels) will reduce over time, and across dyads, following the introduction of the ABC intervention to show a more normative diurnal cortisol rhythm.

Furthermore, the study sought to understand the social validity of the intervention programme as perceived by the parents and intervenors.

## Materials and Methods

### Design

The study followed a mixed methods single case research approach (Onghena et al., 2018). A concurrent, multiple-baseline single-case design was applied across three mother-child dyads, followed by in-depth interviews with parents and intervenors. The combination of experimental control, randomisation, and blinding in this design allows conclusions about effects among subjects to be drawn with high internal validity (Kratochwill and Levin, 2010). Applying Koehler and Levin’s (1998) regulated randomisation procedure, each dyad was randomly assigned—by an independent assistant—to a set of two non-overlapping a priori intervention starting points. In a further step, the specific starting point within each set was also randomly assigned resulting in the final allocation of a five-, eight- or nine-week baseline to each dyad, respectively. Repeated measures of the dependent variables occurred on a weekly basis during the pre-intervention baseline phase (A1), intervention phase (B) and brief post-intervention phase (A2), with some exceptions due to practical constraints (illness, work commitments, or vacation). During Phase B, the ABC intervention was introduced once-weekly for a set 10 sessions per dyad. The observer, dyads and intervenors could not be blinded to phase. All raters, however, were fully independent and blinded to the study phase associated with each observation by randomising the chronological order and anonymising the labelling of video-recordings.

### Participants

#### Mother-child Dyads

The average age, in years, of the mothers at the commencement of the study was 37 (SD: 1.73). Two were first-time mothers and all were the biological mothers of the participating child and were married to the child’s father. All had completed a Bachelor’s-level university degree and were employed on a permanent, full-time basis. Two of the mothers were Black-African, and one was White. The average chronological age, in months, of the children was 25.67 (SD: 11.02), and the average estimated developmental age, in months, was 14.9 (SD: 10.27). All three children were female and obtained Adaptive Behavior Composites on the Vineland Adaptive Behavior Scales ranging between 35 and 71 (Mean: 57.33; SD: 19.5) and had confirmed developmental delays. Two of the children were taking medication at the time of the study—one for seizure control (Carbamazepine, Phenobarbital and Levetiracetam), and the other for the management of challenging behaviours (Risperdal).

Eligible dyads were referred to the study primarily via a centre providing allied health services to infants up to 36 months of age diagnosed with developmental delays and disabilities. This was supplemented by referrals via a private Facebook group for local mental health professionals. The principal investigator (PI) made telephonic contact with the referred dyads to provide further information and schedule the initial screening for developmental age. Dyads were informed that their further participation in the study was contingent on the outcome of the screening assessment.

Adult familial and non-familial caregivers were eligible for inclusion in the study if they lived with, and were the primary caregiver of, the referred child. Children were eligible if they had an estimated developmental age of up to 36 months with an established diagnosis, by a trained health/mental health professional, of either developmental delays or intellectual disability. Dyads were ineligible for participation if either caregiver or child required extended hospitalisation/institutionalisation, if the child had an estimated developmental age significantly outside the range of 6-36 months, or if the child was formally diagnosed with a known genetic syndrome (e.g. Fragile X) in order to ensure that their behavioural phenotypes would not interfere with the assessment of attachment.

#### Intervenor selection

Two intervenors were randomly selected by the PI from a pool of six candidates screened by the developers of the ABC. Both were experienced psychologists with master’s-level qualifications and post-master’s in-service training, and one was pursuing a PhD in Psychology. Training in the ABC protocol, conducted by the Attachment and Biobehavioral Catch-Up Laboratory (University of Delaware, USA), took place in South Africa over two days for the intervenors and research team.

### Context and Setting

The study was conducted in Johannesburg and Pretoria, South Africa, and procedures took place in the homes of the dyads. Observation-based procedures were focussed on naturalistic interactions, hence no special arrangements or equipment—other than video-recording equipment—were required. The observer (the PI) remained in the room during observations to re-angle and pan the camera, when necessary, due to the activity of the dyad.

### Approvals and Consent Procedures

Ethical clearance for the study was obtained from the Health Research Ethics Committee at North-West University, South Africa (NWU-00012-18-A1). Written and verbal consent was obtained from the parent-participants for their own and, separately, their child’s participation.

### Measures

#### Screening

Children’s developmental age was screened using the Vineland Adaptive Behavior Scales, 3^rd^ edition (VABS-3; Sparrow et al., 2016). The VABS-3 produces a standardised total Adaptive Behavior Composite as well as age equivalence estimations for its three functional subdomains—communication, daily living skills and socialisation—and is regularly used to support diagnoses of intellectual disabilities.

#### Maternal Sensitivity

Maternal sensitivity was assessed using the Ainsworth Maternal Sensitivity Scale (AMSS; Ainsworth et al., 1991), which rates the mother’s ability to perceive the child’s signals accurately, and to respond to these appropriately and timeously. A total of 52 home-based naturalistic observations, conducted weekly over a six-month period and lasting one hour each, were video-recorded during all study phases across dyads. The recordings were later independently rated on a nine-point scale ranging from *highly insensitive* (1) to *highly sensitive* (9). Dyads were instructed to interact with one another as they normally would and to disregard the observer and equipment. An independent consultant trained in the AMSS watched the one-hour recordings and selected a 10- to 20-minute segment from each that was suitable for rating sensitivity. Two independent raters (master’s-level students at North-West University, South Africa) were trained, and certified reliable, by an expert in the AMSS (M.J. Bakermans-Kranenburg, Vrije Universiteit Amsterdam, the Netherlands) and subsequently carried out the rating. All 52 video segments were double-coded, showing moderate inter-rater reliability (intraclass correlation coefficient [ICC]: .63; Koo and Li, 2016). Analyses were performed on the mean of both raters’ sensitivity scores at each measurement point.

#### Attachment Security

Attachment security was assessed using the Attachment Q-sort (AQS, version 3; Waters, 1987), a continuous measure of attachment, which contains 90 items describing secure base behaviours of children up to five years of age applied to naturalistic observations of parent-child interactions. These items were sorted by two trained, independent raters into nine piles of 10 items each ranging from *very much unlike the child* (Pile 1) to *very much like the child* (Pile 9), and assigned a score between 1 and 9, accordingly. The observer rating was subsequently correlated with the rating, by experts, of an ideally secure child, producing a score between −1.0 (a child least resembling security) and +1.0 (a child most resembling security). AQS scores above 0.3 have been proposed as cut-off for secure attachment (Solomon and George, 2016). The same recordings were used to rate maternal sensitivity and attachment security although the full hour was used for rating the AQS. The first rater (a master’s-level research assistant with experience in AQS rating), based at the Vrije Universiteit Amsterdam, the Netherlands, rated 41% of the total videos and trained the second rater (a PhD student at the University of the Witwatersrand, South Africa). The second rater sorted all 52 videos and moderate to good inter-rater reliability (ICC: .75) was established on the 41% of double-coded videos. Analyses were performed on the scores of the second rater.

#### Affect regulation

Affect regulation was assessed by means of measuring the diurnal rhythm of cortisol production in the child-participants through saliva sampling. Parents were trained by the PI to collect saliva samples from their child at three equally-spaced time points during the day (Gunnar and White, 2001)—morning (07:30), afternoon (13:30) and evening (19:30)—on one day per week for the full duration of the study. Parents were instructed not to take samples within 30 minutes of the child having been fed or having brushed their teeth, and to delay sampling by a day or two if the child was ill.

Parents were instructed to place a *SalivaBio* Children’s Swab (Salimetrics, LLC) into their child’s mouth for 60-90 seconds, until saturated, before depositing it in a pre-labelled storage tube and storing it in their home freezer. The PI collected frozen samples weekly to bi-weekly and delivered these to a private commercial laboratory where they were stored at −20°C before being thawed, centrifuged and analysed using Liquid Chromotography-tandem Mass Spectrometry (LC-MS). Samples were analysed in smaller batches due to limited laboratory storage. Ten of the 165 samples collected yielded insufficient saliva, resulting in 155 valid samples across dyads. Inter- and intra-assay coefficients of variance were not available from the laboratory.

***Cortisol Data Preparation***. The three raw nmol/L values at each measurement point were reduced using Area Under the Curve (AUC) formulae proposed by Pruessner and colleagues: AUC with respect to Ground (AUC_G_)—overall magnitude of cortisol—and AUC with respect to Increase (AUC_I_)—change in cortisol over the course of the day with reference to the morning value (Pruessner et al., 2003). Decreasing values on these indices are indicative of improvement, with decreasing AUC_G_ values reflecting a lowering of the overall magnitude of cortisol and negative AUC_I_ values suggestive of lower cortisol levels later in the day compared to the morning (i.e. download diurnal slope). Values for eight of the 10 invalid samples were imputed by linear interpolation using the *imputeTS* package (version 3.1) for *R*. The two remaining values occurred at the same measurement point for a single participant, precluding imputation. Furthermore, AUC_G_ and AUC_I_ outliers for all cases were identified using Tukey’s Fence Method (Jones, 2019), resulting in the removal of three AUC_G_ and eight AUC_I_ data points across cases.

#### Social Validity

An adapted version of the Social Validity Scale (SVS; Seys, 1987) asked parents to evaluate the social and practical significance of the intervention upon its completion. The scale consisted of 21 questions pertaining to the intervenor, the number of sessions, the perceived changes due to the intervention, and the techniques used in sessions, amongst others, rated along a five-point scale.

#### Individual Interviews

Individual semi-structured interviews were conducted with the parents prior to, and upon conclusion of, the intervention phase of the study. The first interview—a brief clinical interview—was conducted by the PI, a registered clinical psychologist, at the commencement of the study to gather pertinent historical information on the dyad. The second interview was conducted by an independent master’s-level research assistant at post-intervention with parents and intervenors and ranged from 30 to 60 min. In addition to social validation (e.g. Can you tell me what, for you, was most beneficial about the ABC?; Can you tell me about what you did not find useful about the ABC?; What was your experience of the parent coach?), the parent interviews explored their perceptions of changes in their child and themselves due to the intervention and the intervenor interviews explored their perceptions of how the dyads responded to the intervention. Interviews were audio-recorded with a digital recorder and subsequently transcribed, verbatim, for the purposes of analysis conducted by the PI and an independent second coder (a master’s-level research assistant based at the Vrije Universiteit Amsterdam, the Netherlands).

### Intervention

The Attachment and Biobehavioral Catchup intervention was originally developed for infants who have experienced neglect and maltreatment but deemed potentially valuable for parents of children with ID or developmental delays. The intervention consists of a fixed set of 10 sessions implemented in the home of the dyad on a weekly basis, for one hour, by a ‘parent coach’. Although the intervention is manualized (Dozier et al., 2016), each session is tailored to the circumstances of the specific dyad.

The first two sessions highlight the importance of nurturing behaviours. Here, parent coaches assist parents in recognising that their children need them even in the absence of a clearly identifiable signal of distress. Sessions three and four foreground synchrony which is facilitated through helping parents recognise and understand the importance of following the child’s lead. Sessions five and six focus on helping parents interact in nonintrusive and nonfrightening ways. Sessions seven and eight turn towards the concept of ‘voices from the past’ in which parents consider how their own issues from the past can affect their parental behaviour. The last two sessions serve as opportunities to consolidate the lessons learned over the previous sessions and to celebrate changes that have occurred.

Although the primary caregiver and child are both required to be present during the intervention session, the intervention is parent-focussed with the parent coach avoiding direct engagement with the child. During sessions, parent coaches deliver session content (such as research evidence) relevant to the session theme while simultaneously responding to the real-time interactions between the parent and child using ‘in-the-moment’ commenting. This is supplemented by video-feedback using moments of parent-child interaction video-recorded during the previous session as well as homework tasks aimed at reinforcing skills. During sessions, parent coaches pay close attention to encouraging parents’ sensitivity by spotlighting even subtle instances of target parenting behaviours. During later sessions, parents’ attention is gently directed to the child’s signals that go unnoticed by the parent. In the current study, all dyads completed the full 10 sessions over a period ranging 16-17 weeks with an average session duration of 48.43 minutes (SD: 5.15).

#### Treatment fidelity

ABC is a fully manualised intervention, in which intervenors received training. Case supervision—using video-recorded sessions—occurred weekly over an eight-month period. The supervisor randomly selected a five-minute segment of the recording which was coded for on-target in-the-moment comments. Intervenors were expected to make an average of one on-target in-the-moment comment per minute to meet adherence criteria (Caron et al., 2016). To qualify for certification, at least 80% of in-the-moment comments had to be on-target, and at least one information component included in the comment, on average (Caron et al., 2016). Both intervenors in the current study demonstrated adherence to fidelity criteria (see Supplemental Material-Table S1).

### Data Analysis

#### Visual Analysis

Visual analysis was conducted using the *SCVA* package (version 1.3.1; Bulté and Onghena, 2012) for *R* (version 4.0.2) to assess the performance of the dyads. Data were inspected for mean level shift, split-middle trend, variability and immediacy or latency of change (Gast and Spriggs, 2010). Change in level was assessed by comparing the mean performance on the dependent variables between phases. Relative change between the baseline and intervention phase (Mdn_Intervention[1st half]_-Mdn_Baseline[2nd half]_) was assessed to determine the immediacy or latency of intervention effect. Trend was used to evaluate the direction of change within each phase over time. Change in trend was then determined by comparing trend lines between adjacent phases. Change in the upward direction was deemed improvement for maternal sensitivity and attachment security, while change in the downward direction was deemed improvement on the cortisol indices.

#### Statistical Analyses

**Randomisation Testing**. Additionally, randomisation tests for multiple-baseline designs were performed using the *SCRT* package (version 1.3.1) for *R* to assess the difference in mean performance between phases on the outcome variables across subjects (Bulté and Onghena, 2009). Phases B and A2 were expected to be superior to Phase A in terms of the dependent variables. Randomisation testing was performed on the raw data for both maternal sensitivity and attachment security, and on the two derived summary statistics (AUC_G_ and AUC_I_) for the cortisol data. Statistical significance was determined at *p* < .05.

**Trend Estimation**. Exploratory statistical estimation of the trend within each phase was carried out using the non-parametric Theil-Sen estimator (Wang and Yu, 2005). Insensitive to outliers, the Theil-Sen estimator represents the median of all regression slopes for all pairs of data points within each phase, offering a statistical estimation of the trend within the respective phases. This was done using a web-based Theil-Sen Calculator (version 2.0) via the www.singlecaseresearch.com website (Vannest et al., 2016).

**Slope and Level Change**. The Slope and Level Change (SLC) procedure outlined by Manolov et al. (2016) was applied, using the *SLC* package (version 0.3) for *R*, in instances of notable pre-intervention improvement, to control for the influence of improving baseline trend on the detection of an intervention effect during Phase B.

**Overlap**. Furthermore, to measure the size of intervention effect, the Nonoverlap of All Pairs (NAP) was calculated using the web-based application of the *SingleCaseES* package (version 0.5; Pustejovsky and Swan, 2018) for *R*. The NAP provides an indicator of performance differences for each case between phases and provides insight into the extent to which data in the baseline versus intervention phases do not overlap. It is “the probability that a score drawn at random from [the] treatment phase will exceed (overlap) that of a score drawn at random from [the] baseline phase” (Parker and Vannest, 2009: 359). A greater degree of nonoverlap is an indicator of greater performance change and hence a stronger effect. NAP values range between 0 and 1, with values 0.5 and over indicative of improvement. The NAP is an accepted indicator of the magnitude of performance change in single-case designs, offering a superior index of effect size compared to other overlap indices (Parker and Vannest, 2009).

#### Qualitative Analysis

Post-intervention interviews were analysed qualitatively—via *Atlas.ti* (version 8 for Windows)—using inductive thematic analysis to identify themes (Braun and Clarke, 2006). Two coders separately organised their respective codes into preliminary themes and subthemes which were then discussed to reach broad agreement on the coding scheme and preliminary themes. Based on this discussion, revisions were made to the coding and thematic organisation followed by a final meeting between the coders to confirm and finalise the thematic areas.

## Results

### Effects on Maternal Sensitivity

**Figure 1.**
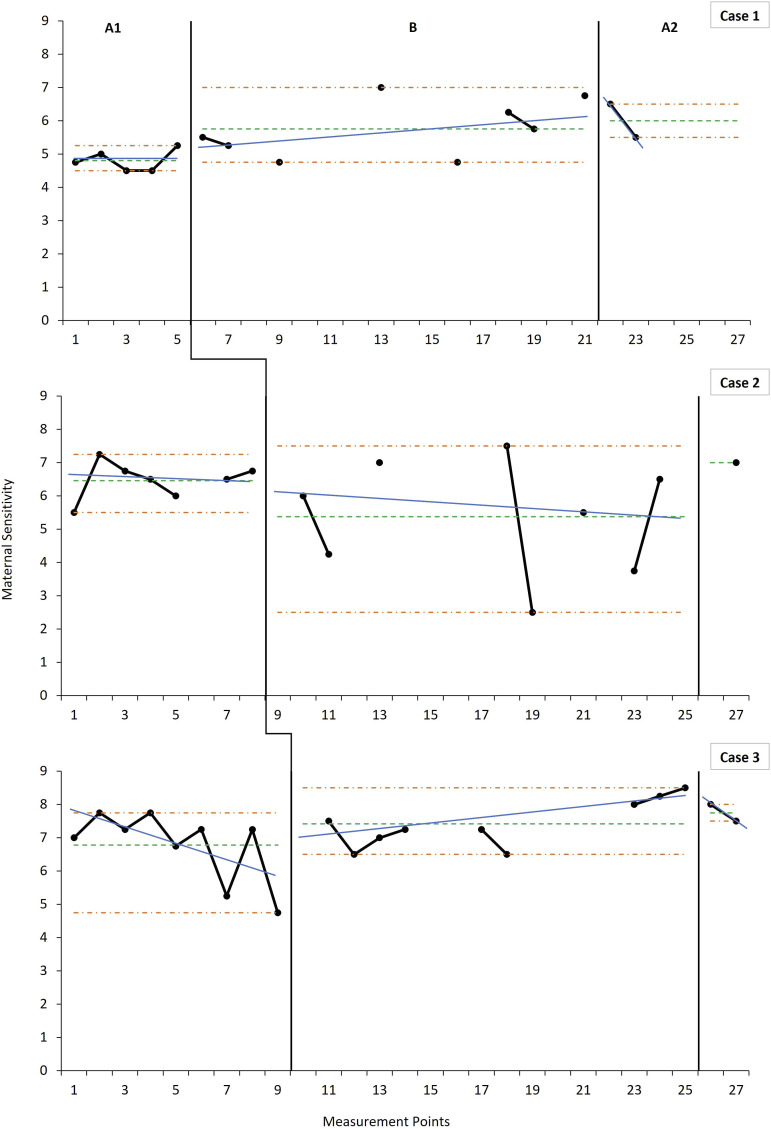
Weekly measurements of maternal sensitivity across phases indicating level and trend estimates. *Note:* For the baseline (A1), intervention (B) and post-intervention (A2) phases, the horizontal parallel dashdot lines (orange) denote the range as an indicator of variability, the singular dashed horizontal line (green) indicates the mean level, and the solid angled line (blue) indicates the split middle trend estimate. Scores on the Ainsworth Maternal Sensitivity Scales range from 1 (highly insensitive) to 9 (highly sensitive). Data points not connected with solid black lines are due to missing measurements that occurred during the course of data collection.

Mean maternal sensitivity (see Figure 1) improved from baseline to post-intervention across all cases (see Supplemental Material-Table S2, for mean level and mean change values). Case 2 was the only case in which mean maternal sensitivity decreased from baseline to intervention, although there was comparatively greater variability for Case 2 during the intervention phase. In addition, Cases 1 and 3 demonstrated immediacy of effect while Case 2 showed latency. Cases 1 and 3 showed a change in trend towards acceleration from baseline to intervention, suggesting improving maternal sensitivity over time following the introduction of the ABC. Although for Cases 1 and 3 there was a slight deceleration post-intervention, the overall post-intervention mean exceeded mean baseline sensitivity. In contrast, Case 2 demonstrated a slight decelerating, contra-therapeutic trend following the introduction of the intervention, although the variability in the data for this case precludes a confident determination of trend in the intervention phase.

The greatest nonoverlap was evident across phases and dyads for maternal sensitivity (See Table 1 for NAP indices). Although only Case 1 showed a medium effect from baseline to intervention (compared to weaker effects in Cases 2 and 3), the remaining two cases showed a medium effect from baseline to post-intervention. Table 2 presents results from the randomisation testing which showed that the phase effect was not statistically significant across the three cases on maternal sensitivity (*p* = .21), and no additional statistically significant changes in slope were identified from the exploratory Theil-Sen estimations (see Supplemental Material-Table S3).

**Table 1.** Nonoverlap of All Pairs (NAP) across study phases for maternal sensitivity, attachment security and cortisol indicators.

	A1-B				A1-A2				B-A2			
	NAP	SE	95% CI		NAP	SE	95% CI		NAP	SE	95% CI	
			Lower	Upper			Lower	Upper			Lower	Upper
Sensitivity
Case 1	.86	.10	.53	.97	1.00	0	1.00	1.00	0.59	0.21	0.22	0.88
Case 2	.32	.15	.13	.61	.86	NA	.29	.99	0.81	NA	0.26	0.98
Case 3	.65	.13	.38	.84	.89	.11	.43	.99	0.67	0.17	0.27	0.91
Security
Case 1	.86	.09	.54	.97	1.00	0	1.00	1.00	0.69	0.31	0.28	0.92
Case 2	.52	.16	.26	.77	.43	NA	.09	.86	0.38	NA	0.07	0.83
Case 3	.62	.14	.36	.82	.61	.16	.23	.89	0.39	0.16	0.11	0.77
Cortisol Magnitude (AUC_G_)
Case 1	.36	.15	.14	.66	.20	.20	.03	.67	0.39	0.20	0.11	0.77
Case 2	.38	.19	.16	.67	.50	.22	.15	.85	0.55	0.16	0.20	0.85
Case 3	.69	.13	.42	.87	.50	.50	.17	.83	0.35	0.35	0.10	0.74
Cortisol Change (AUC_I_)
Case 1	.72	.17	.41	.90	.40	.24	.10	.80	0.05	0.05	0	0.51
Case 2	.29	.20	.09	.64	.75	.25	.28	.96	1.00	0	1.00	1.00
Case 3	.44	.15	.22	.69	.13	NA	.01	.70	0.30	NA	0.05	0.79

*Note:* 0-.65 = weak effects; .66-.92 = medium effects; .93-1 = strong effects (Parker and Vannest, 2009). NA = No available SE due to only one data point at post-intervention (A2).

**Table 2.** Results of randomisation testing for maternal sensitivity, attachment security and cortisol indicators.

	Including A2		Excluding A2	
	Observed Statistic^a^	*p*	Observed Statistic^a^	*p*
Sensitivity	.28	.17	.19	.21
Security	.14	.15	.14	.23
Cortisol Magnitude (AUC_G_)	−.8	.46	−2.03	.46
Cortisol Change (AUC_I_)	.09	.42	−.23	.44

*Note:* Randomisation tests for multiple baseline designs conducted in *R* are only possible with two-phase AB studies. As such, due to the addition of an A2 phase, the randomisation tests in the present study were conducted twice—including and excluding A2 phase data points as part of the B-phase data—in order to assess the impact of the A2 data on the overall significance of the results.

^a^Test statistic for security and sensitivity = B-A; test statistic for cortisol variables = A-B.

### Effects on Attachment Security

**Figure 2.**
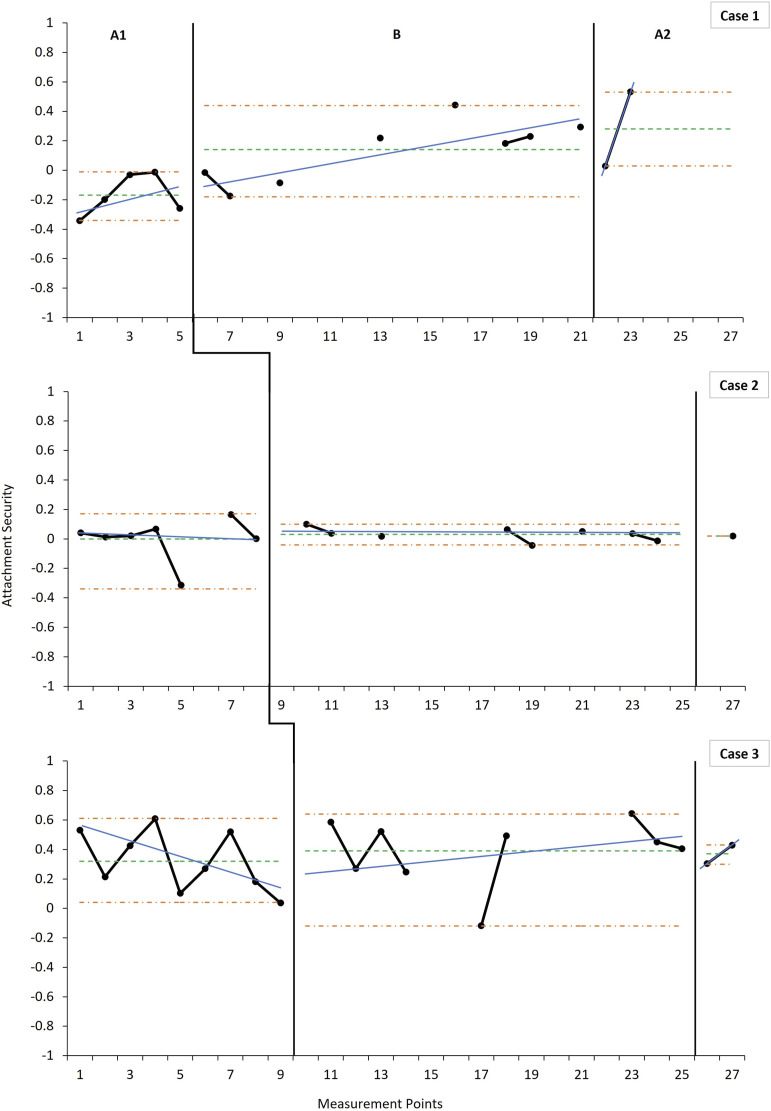
Weekly measurements of attachment security across phases indicating level and trend estimates. *Note:* For the baseline (A1), intervention (B) and post-intervention (A2) phases, the horizontal parallel dashdot lines (orange) denote the range as an indicator of variability, the singular dashed horizontal line (green) indicates the mean level, and the solid angled line (blue) indicates the split middle trend estimate. Scores on the Attachment Q-Sort take the form of a correlation co-efficient, ranging from −1 to 1; with 1 representing a child highly similar to a prototypically secure child and −1 representing a child highly dissimilar to a prototypically secure child. Scores above 0.3 are indicative of secure attachment. Data points not connected with solid black lines are due to missing measurements that occurred during the course of data collection.

Mean attachment security (Figure 2) improved across all three cases from baseline to intervention, with Case 1 showing the largest change in average security across phases, and Case 2 the smallest. Relative change showed an immediate intervention effect in all cases, although smaller in Case 2 and 3 compared to Case 1. The improvement in mean security was maintained at post-intervention for all three cases, despite negligible declines from intervention to post-intervention for Cases 2 and 3. Both Cases 1 and 3 showed an improving trend during the intervention phase following the introduction of the intervention, even after controlling for initial baseline improvement in Case 1. Trends in Case 2 were negligible across all phases with no clear improvement or decline in attachment security over time.

 Case 1 demonstrated medium to strong effects across phases for attachment security with the remaining two cases showing consistently weak effects (Table 1). The phase effect was not statistically significant on the randomisation test across the three cases on attachment security (*p* = .23) and exploratory Theil-Sen estimations revealed no additional statistically significant changes in slope on this variable.

### Effects on Cortisol Regulation

**Figure 3.**
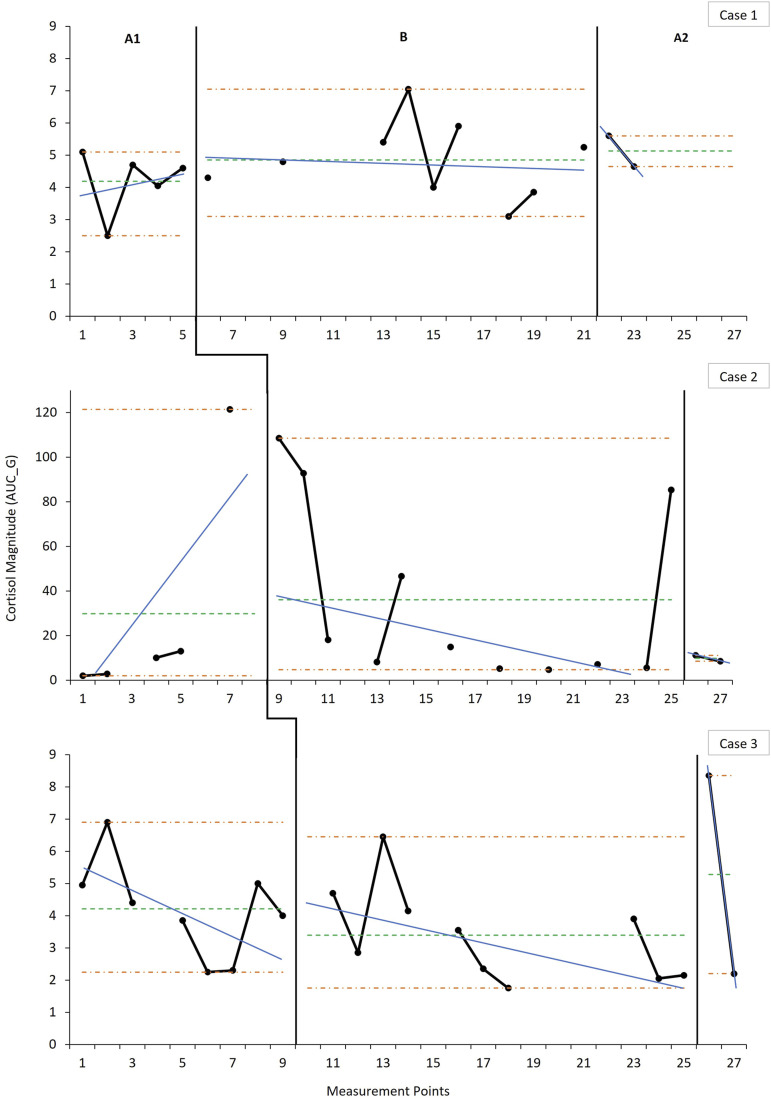
Weekly measurements of the overall magnitude of cortisol production over the day across phases indicating level and trend estimates. *Note:* Magnitude is measured over the course of 12 h (7:30–19:30). For the baseline (A1), intervention (B) and post-intervention (A2) phases, the horizontal parallel dash-dot lines (orange) denote the range as an indicator of variability, the singular dashed horizontal line (green) indicates the mean level, and the solid angled line (blue) indicates the split middle trend estimate. Data points represent the AUC_G_ values calculated for each measurement point using the three cortisol daily readings (7:30, 13:30 and 19:30). Data points not connected with solid black lines are due to missing measurements that occurred during the course of data collection. Due to the large variability in the individual data series for Case 2 with regards to the AUC_G_, the *y*-axes were not standardised across cases in order to facilitate visual inspection and analysis.

**Figure 4.**
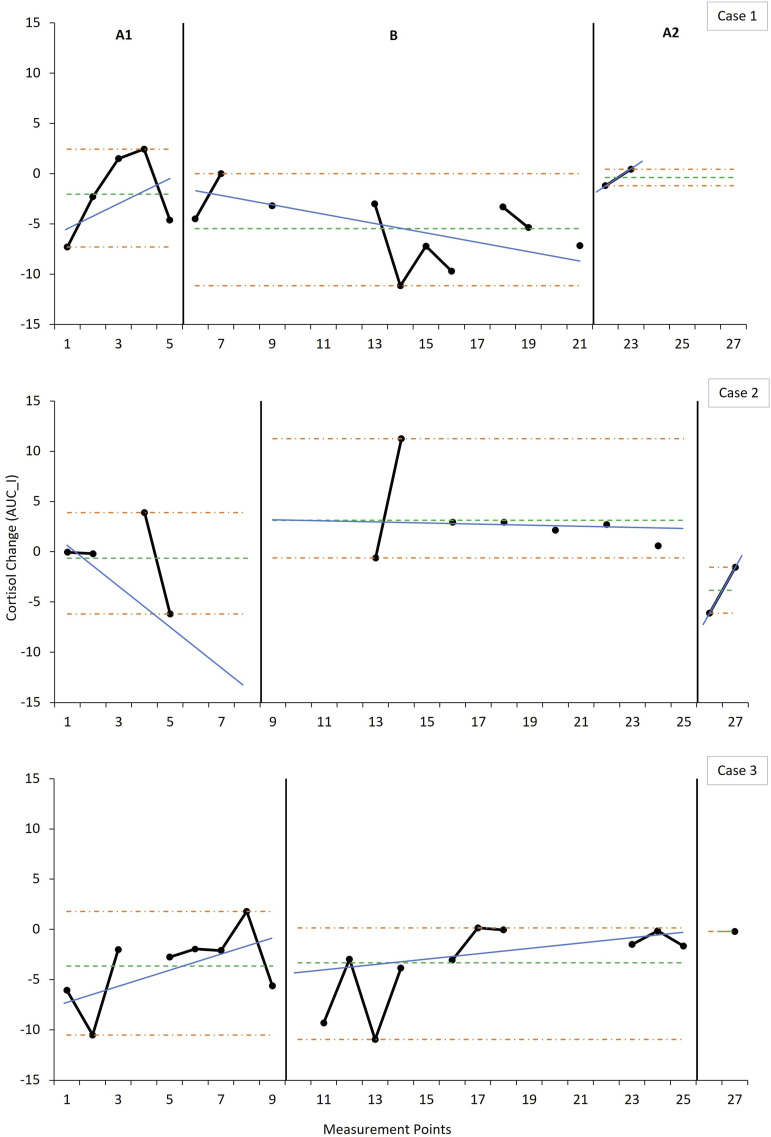
Weekly measurements of the change in cortisol production over the day across phases indicating level and trend estimates. *Note:* Change is measured over the course of 12 h (7:30–19:30). For the baseline (A1), intervention (B) and post-intervention (A2) phases, the horizontal parallel dash-dot lines (orange) denote the range as an indicator of variability, the singular dashed horizontal line (green) indicates the mean level, and the solid angled line (blue) indicates the split middle trend estimate. Data points represent the AUC_I_ values calculated for each measurement point using the three cortisol daily readings (7:30, 13:30 and 19:30). Data points not connected with solid black lines are due to missing measurements that occurred during the course of data collection.

Data on cortisol regulation (Figure 3 [AUC_G_] and Figure 4 [AUC_I_]) did not clearly demonstrate a consistent pattern of change across cases. Each case showed a distinct intra-case pattern across all phases on both indicators along with notable variability, precluding reliable estimations of level and trend. However, Case 1 demonstrated a discernible shift in AUC_I_ level and trend in the expected direction during the intervention phase, but not in AUC_G_. This improvement in AUC_I_ was, however, not sustained at post-intervention and did not replicate in the other cases. Case 2 showed relatively large overall cortisol elevations and significant periodic spikes, demonstrated in the comparatively and notably larger and more variable AUC_G_, and positive AUC_I_, values across phases. Although Case 3 showed no notable average change in AUC_I_ during the intervention phase, data indicated an early improvement, and later decline, reflected in the accelerating trend. The inverse pattern was observed in AUC_G_ values for Case 3—early increase, and later decrease, with decelerating intervention phase trend. It is noteworthy, however, that the average AUC_I_ for both Cases 1 and 3 were in the negative range across phases (Figure 4).

Overall, there was a small degree of nonoverlap on the cortisol indicators, suggesting a weak effect of the intervention on cortisol production. Furthermore, randomisation testing showed that the phase effect was not statistically significant for either cortisol indicator (AUC_G_: *p* = .46; AUC_I_: *p* = .44) and no additional statistically significant changes in slope were identified.

### Parents’ and Intervenors’ Evaluations of the Intervention

All three parents reported changes in their children over the course of the intervention. Themes that were distilled from the interviews included greater initiative-taking and confidence in the children, which parents attributed to them allowing the child to take the lead. Parents also noted more positive parent-child interactions and greater engagement and openness to interaction on the part of their children, including more concerted attempts at seeking physical closeness. This was exemplified by one parent who commented on how “[my child] became more open…[she would] come more often to [me]…because [she] know[s], okay, mommy will respond” (Parent, Case 1).

Themes regarding changes in the parents themselves were focussed around the three core intervention targets. Parents reported having learned how to better align with their child. One parent commented, in relation to her daughter, that “even though I am attentive of her…I’ve sort of let her be herself as well. I let her to be herself instead of trying to push too hard to get her to what I want her to do” (Parent, Case 2), which is indicative of having learned how to follow the child’s lead. Parents also commented on having learned the importance of behaving in ways that make their children feel better when they are distressed even when this did not—at first—necessarily come naturally, as exemplified by a parent who commented on how “[when she’s upset] I’m not just leaving her to get on with it. I’m bringing the love back there. I’m sitting there saying ‘I’m still here for you’” (Parent, Case 3), suggesting the adoption of more nurturing responses when the child was upset. Parents also took note of increasingly coming to notice and understand the necessity of reducing frightening behaviours. One of the parents, reflecting on what she had learned about herself, stated that “being scary is one of them. And I’ve noticed how that affects [my child]…if I raise my voice for anything, she will just sit there and she looks at me deer-in-headlights…and goes and has a good cry. So I’ve really got to get to the point where it’s not shouting [anymore]…” (Parent, Case 3).

In addition to changes in relation to the three core intervention targets, parents also noted changes with regards to another session theme—coming to recognise voices from the past. One mother noted how she often would use a serious and irritated tone in response to her child crying, instead of a more gentle, nurturing and curious tone but “after a while when we were doing our [ABC] sessions, I actually realised I respond based on…voices from the past” (Parent, Case 2). Lastly, parents highlighted the manner in which they had, over the course of the intervention, improved in their observation, understanding and responsiveness regarding their children’s cues as reflected in the following extract:

…she’s [premature], so it was very difficult for me to understand her…she’s this quiet child, sometimes…she’s not happy about something, [but] she’ll be quiet and I think you know she’s fine, but then now I can observe her and then I’ll take her and then hold her and then she becomes like ‘okay, mommy’s here’, then she becomes happy (Parent, Case 1).

All three parents experienced the intervention as beneficial and relevant, and felt that it could be of benefit to others who have a child with developmental delays. On the SVS, parents also rated the intervention favourably. Parents rated positively the impact of the intervention on changes in their parenting behaviours and in their children, as well as on their feelings of competence as a parent and parenting stress. All parents rated the intervention as “Very Good” and would recommend it as such to others because, in their opinion, it was effective at improving their relationships with their children.

Intervenors highlighted change in child behaviour and change in the child’s expectations of the parent. Themes in the interviews regarded increased initiation of behaviours (“it was as if [the child] came alive”—Intervenor, Case 1), greater intentionality in seeking proximity on the child’s part (“you could see…the difference in the kid, the way that she was responding to mom and leaning into her”—Intervenor, Case 3), and increasingly positive parent-child interactions (“she was safe where in the beginning it was…it really looked like a child that was just totally lost”—Intervenor, Case 2). Particularly noteworthy were the intervenors’ observations related to changing expectations of care from the child based on shifting parenting behaviours such as following the lead or responding to signals (“she was eventually having an expectation of seeing her mom respond”—Intervenor, Case 1).

Both intervenors also highlighted notable changes in the parents related to particular core intervention targets. One such change pertained to enhanced parent-child synchrony that emerged as the result of improvements in parents following the child’s lead, which served to encourage parents when they observed positive responses from the child. One intervenor commented on how the parent with whom she was working “was beginning to initiate less and allowing [the child] to initiate more…mom was encouraged to follow more, which then also encouraged [her child] to lead more and then mom follows more” (Intervenor, Case 1). Nurturance was the second core target highlighted by intervenors. They observed that the parents increasingly came to understand the importance of prompt, nurturing responses and were able to put this understanding in practice, resulting in observable changes in child behaviours, as noted in the following reflection:

Mom went from ‘…I don’t know what she wants’…[to] ‘ah, you want some food. Ah, your tummy’s full’. It’s [like] day [and] night…and you could see…the difference in the kid, the way that she was responding to mom and leaning into her…(Intervenor, Case 2).

Although the intervenors had some early reservations related to the applicability of the intervention for children who were lower functioning, after noting improvements in the dyads over time intervenors echoed parents’ support for the intervention’s benefit and relevance to families with a child who has development delays or intellectual disability.

## Discussion

Overall, visual analysis supported the feasibility, use and utility of the ABC in shifting maternal sensitivity (Hypothesis 1) and attachment security (Hypothesis 2) in individual families with children with intellectual disabilities and developmental delays. This was complemented by the experiences of the parents and intervenors who noted positive changes in parent and child behaviours over the course of the intervention. Visual analysis also provided a first implementation of observer-rated sensitivity and attachment security to track changes from baseline to intervention and post-intervention. However, statistical tests of the time series were inconclusive. The findings related to affect regulation (Hypothesis 3) also showed no clearly replicable evidence of an intervention effect.

Patterns of change in maternal sensitivity were observed across all three dyads. Two cases demonstrated improvements in maternal sensitivity following the introduction of the ABC while the third case showed an improvement later, at post-intervention. The improvement in sensitivity was as expected given that the ABC is parent-focused and is aimed, ultimately, as enhancing sensitive responsiveness in the parent. The current findings on sensitivity are encouraging and correspond with previous studies which have found significant intervention effects on sensitivity in groups of parents who received the ABC versus a control condition (Berlin et al., 2018; Bick and Dozier, 2013; Hepworth et al., 2020; Yarger et al., 2020). Mean sensitivity levels in the present study also meet or exceed the mean AMSS-determined sensitivity recently reported in a sample of South African mothers with typically developing children (Dawson et al., 2021). The parents in the current study were, on average, never—even at baseline—within the insensitive (1-4) range, which also corresponds with the findings of a study by Feniger-Schaal and Joels (2018) among parents of children with intellectual disabilities. Zevalkink and Riksen-Walraven (2001) found that sensitivity was higher in Indonesian mothers who had a higher household income, higher level of education and husbands who had similar, much like parents in the current study. It is possible, therefore, that the study may not be fully representative of the sociodemographic profile of parents in greatest need in South Africa.

It is encouraging, however, that despite already sensitive baseline levels, the parents in the present study continued to improve in their sensitive responsiveness. In the case that did not show initial improvement (Case 2), the variability in the intervention phase data clouds reliable estimation of changes in level and trend. Despite a mean decline in sensitivity of over 1 scale point, the sensitivity rating remained within the sensitive range (5-9). Although there was seemingly a downward trend in sensitivity at post-intervention, mean sensitivity remained above baseline levels. This downward trend may be accounted for by the cessation for parents of intensive, weekly feedback on their interactions with their child, resulting in the drop-off. This suggests that periodic follow-up sessions may be required as a refresher for parents. However, given that the A2 phase was constituted of only two measurement points, additional data is required to confirm a sustained downward trend.

Observed changes in sensitivity were echoed in parents’ and intervenors’ subjective reports of the development and mastery of parenting capacities as a mother of a child with significant developmental challenges, resulting from the intervention. This shift may be related to the learning and personal mastery of parenting challenges that took place during the intervention procedures (Bandura, 1977). This is consistent with Platje et al.’s (2018) findings that the VIPP-V intervention enhanced parenting self-efficacy among parents of children with visual and visual-and-intellectual disabilities, even in the absence of observed improvements in parent-child interactions.

The improvements in mean attachment security in Cases 1 and 3 aligned with the improvement in sensitivity (Fearon and Belsky, 2016; Feniger-Schaal and Joels, 2018). The change in Case 3 was more muted possibly due to a ceiling effect (Bakermans-Kranenburg et al., 2003). This muted pattern was also reflected in Case 2 although with mean AQS scores in the insecure range retained across phases. The broad shift towards greater security over time is roughly consistent with previous studies of the ABC which have found positive effects on attachment (Bernard et al., 2012; Dozier et al., 2009) among children who received the ABC versus controls. Group trials have on average reported modest effect sizes (Bakermans-Kranenburg et al., 2003; Letourneau et al., 2015), and the current data suggest that modest shifts in attachment security may also be observed when tracking individual dyads over the course of intervention.

Parents’ and intervenors’ subjective accounts underscored changes in attachment behaviour, such as increasing proximity-seeking behaviours and greater confidence and initiative-taking in exploring their immediate environments. This points to the practical relevance and social validity of the intervention. These subjective reports also point towards the potential value of monitoring discrete attachment behaviours as potential precursors of shifts in the organisation of attachment behaviour that might be detected with research instruments such as the AQS. Furthermore, parents’ intimate familiarity with their children may enable them to perceive subtle shifts in behaviour that are difficult to detect for raters who are not familiar with the child’s particular signal repertoire (Kruithof et al., 2020; Tessier et al., 2002; Vandesande et al., 2019a). For Case 2, the child’s seizure disorder and relatively lower adaptive functioning may have affected the level and intensity of her proximity- and contact-seeking as well as contact-maintaining behaviours (Vandesande et al., 2019b). It is plausible that the mother may have adapted, over time, to her child’s uniquely expressed signals, hence her suitable level of sensitivity whereas AQS raters—in the absence of this adaptation—may have incorrectly interpreted the fewer and less intense typical attachment behaviours as insecurity. To investigate therapeutic changes in attachment quality over the course of intervention, measures may therefore not only need to be taken repeatedly but also allow for fine-grained analysis of idiosyncratic ways in which children, specifically those with intellectual disabilities or developmental delays, may express their needs and expectations towards their attachment figure.

Little indication was found for an impact of the intervention on affect regulation. Cases 1 and 3 did not demonstrate notable baseline dysregulation in the form of hypercorticolism, instead showing evidence of a normative diurnal pattern (Baumler et al., 2013). Effects of the ABC on cortisol production may be expected, in particular, when affect is dysregulated (Dozier et al., 2008). Parents in the current study were not maltreating parents—in contrast to prior ABC research—and were consistently sensitive even at baseline, and may therefore have already been serving a regulating function (Perry et al., 2014). Although this ceiling effect obscures intervention effects, changes were observable within cases, albeit in divergent ways.

The changes observed in Case 1 reflected a steepening of the diurnal cortisol slope due to increasing morning concentrations (see Supplemental Material-Figure S1) in accordance with hypothesized expectations. This suggests that the child developed improved regulatory capacities (Bradley, 2000; Sterkenburg et al., 2008a, 2008b), corresponding with concomitant improvements in maternal sensitivity and attachment security. Although Case 3 shows a similar initial effect, there was a later decline, contrary both to expectations and to improvements in maternal sensitivity and attachment security. The flattening of the diurnal slope due to lowering morning concentrations corresponds with the suspension of Risperdal use roughly towards the midway point of the intervention. It is possible that the disruption to the antipsychotic medication regimen resulted in a withdrawal response such as disturbances in sleep hygiene (Ruths et al., 2004), which has been associated with low morning salivary cortisol concentrations (Backhaus et al., 2004). Medication could also have played a role in Case 2 whose unusually elevated cortisol levels may be attributed to her use of Carbamazepine, in particular, which has been found to result in cortisol elevations (Cano-Lopez and Gonzalez-Bono, 2019). Furthermore, the erratic spiking above already elevated cortisol could potentially be the result of intermittent, undetected, seizures which have also been associated with cortisol elevations independently from medication (Galimberti et al., 2005). The potential effects of medication in Cases 2 and 3 along with the added effect of undetected seizure activity in the former prevent reliable determination of an intervention effect on cortisol regulation, precluding cross-case replication.

The intervention was also highly regarded and rated by the parents in the study who commented, qualitatively, on the usefulness, value and relevance of the intervention. Fidelity estimations pointed to a high degree of treatment integrity, indicating that the intervention was implemented as intended and consistently over time and across cases. The absence, however, of consistent replication as well as the lack of statistically conclusive tests of the observed effects warrants caution in drawing firm conclusions. The findings of this first study to explore the effects of the ABC in dyads where the child has an intellectual disability or developmental delays does, however, warrant further testing of the benefit at the level of this population.

The current study has a number of limitations. Statistical analysis was limited by the randomisation scheme and the *a priori* determination of baseline lengths, minimising the number and combinations of possible intervention starting points. This, combined with the inclusion of the minimum recommended number of cases, resulted in a limitation in the size of the randomisation distribution, therefore reducing statistical power. Additional cases would also increase replications of possible intervention effects, improving internal validity. In addition, only middle-class families participated in the study. This may have contributed towards the observed ceiling effects given that these families’ social positioning may have served to mitigate against the impact of socioeconomic and other contextual stressors on maternal sensitivity, for example (De Falco et al., 2014). Unlike the families in the current study, many South African families-in-need live in untoward socioeconomic conditions with limited access to support for parents caring for a child with intellectual disability which may compound an already stressful living environment. It is possible that these conditions may produce differential intervention effects and should therefore be factored into future studies of the ABC in South Africa. Mothers were recruited into the study via private practitioners who did not specifically focus on supporting families with children with disabilities who also experience poverty and deprivation. Due to the lack of an integrated system of public health services for young people with intellectual disabilities in South Africa, their dispersed distribution in the population, and barriers such as stigma (Mkabile and Swartz, 2020), future work is needed to find ways of reaching families facing multiple risks.

Another possible limitation relates to the use of the AQS which has not been validated for populations of children with intellectual disabilities (Vandesande et al., 2019a). Microanalytic studies akin to that by Beebe et al. (2010) may be enlightening in this regard. Alternatively, or additionally, to avoid raters’ unfamiliarity with the child and to capitalise on parental knowledge of the child’s idiosyncratic expressions, an alternative approach may be to utilise parent-rated instruments, adopting a simplified AQS to make the rating process easier and less cumbersome, such as the 62-item Attachment Q-List (John et al., 2012). Once-weekly, single-day, saliva sampling is another possible limitation preventing the day-to-day variability in cortisol being accounted for, especially when testing the AUC (Rotenberg et al., 2012; Segerstrom et al., 2014). Future studies may also consider the use of alternative psychophysiological measures to augment or supplement cortisol sampling to manage the potential pitfalls in salivary cortisol measurement such as day-to-day variation or the impact of medication (e.g., Vandesande et al., 2020).

This was the first study to test the ABC in dyads with a child with significant developmental delays or an intellectual disability and to do so at the level of individual dyads over time using observer-rated measures of sensitivity and security. It was also the first such trial to implement the ABC in an African context. The study demonstrated improvements in both maternal sensitivity as well as attachment security over the course of the intervention. Moreover, the intervention was experienced as relevant, useful, and having impacted positively on parent and child behaviours. The study provides the first evidence for the feasibility, use, and utility of the ABC in this population and underscores the relevance of attachment-based interventions, more generally, for this cohort. The findings provide the building blocks for further study of the ABC for those with intellectual disabilities that may facilitate broader community-based implementation in contexts such as South Africa in order to increase access to evidence-based interventions.

## Supplemental Material

Supplemental Material - Using attachment and biobehavioral catch-up with young children with developmental delays: A multiple-baseline trial of attachment, sensitivity, and cortisol Click here for additional data file. Supplemental Material for Using attachment and biobehavioral catch-up with young children with developmental delays: A multiple-baseline trial of attachment, sensitivity, and cortisol by Ahmed Riaz Mohamed, Paula Sterkenburg, Joshua G. Yeatman, Esmé van Rensburg and Carlo Schuengel in Journal of Intellectual Disabilities

Supplemental Material - Using attachment and biobehavioral catch-up with young children with developmental delays: A multiple-baseline trial of attachment, sensitivity, and cortisol Click here for additional data file. Supplemental Material for Using attachment and biobehavioral catch-up with young children with developmental delays: A multiple-baseline trial of attachment, sensitivity, and cortisol by Ahmed Riaz Mohamed, Paula Sterkenburg, Joshua G. Yeatman, Esmé van Rensburg and Carlo Schuengel in Journal of Intellectual Disabilities
