# Supplementary material for: Using Attachment and Biobehavioral Catch-up with young children with developmental delays: A multiple-baseline trial of attachment, sensitivity, and cortisol
Source: J Intellect Disabil. 2022 Jun 1;27(4):847–70. doi: 10.1177/17446295221104614 (PMC10647892; doi:10.1177/17446295221104614)
Supplement: Supplemental Material - Using attachment and biobehavioral catch-up with young children with developmental delays: A multiple-baseline trial of attachment, sensitivity, and cortisol [file sj-pdf-1-jld-10.1177_17446295221104614.pdf]

Supplementary Figure S1

Morning, Afternoon and Evening Raw Score Cortisol Concentrations for Baseline and Intervention

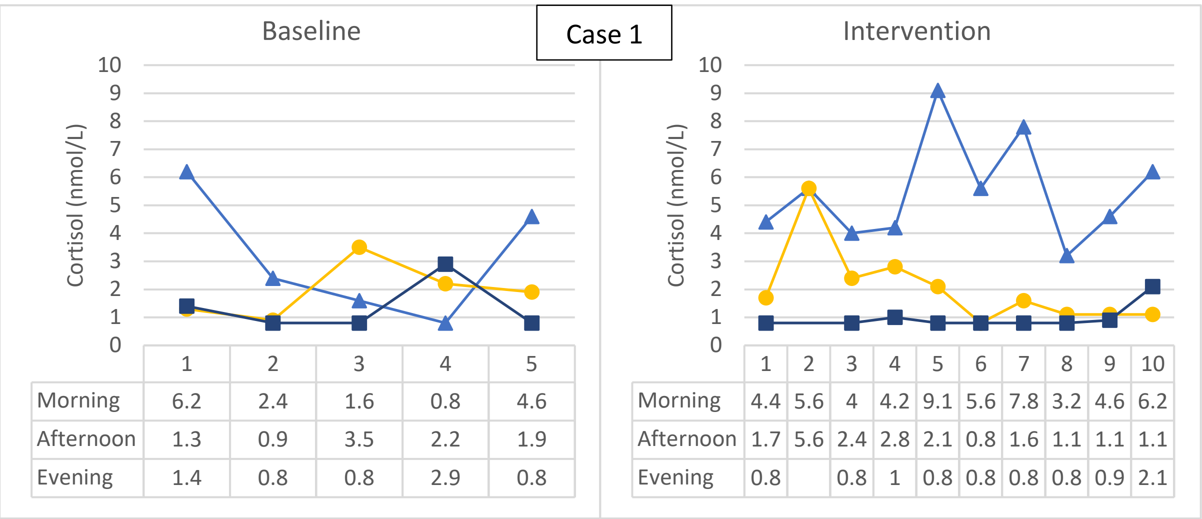

Case 1 – Baseline

	1	2	3	4	5
Morning	6.2	2.4	1.6	0.8	4.6
Afternoon	1.3	0.9	3.5	2.2	1.9
Evening	1.4	0.8	0.8	2.9	0.8

Case 1 – Intervention

	1	2	3	4	5	6	7	8	9	10
Morning	4.4	5.6	4	4.2	9.1	5.6	7.8	3.2	4.6	6.2
Afternoon	1.7	5.6	2.4	2.8	2.1	0.8	1.6	1.1	1.1	1.1
Evening	0.8		0.8	1	0.8	0.8	0.8	0.8	0.9	2.1

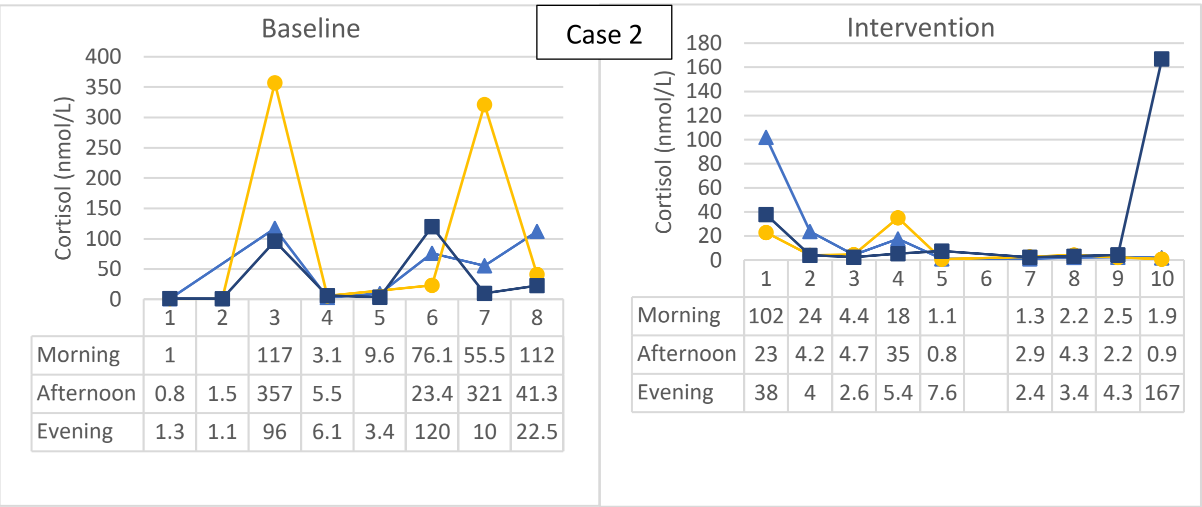

Case 2 – Baseline

	1	2	3	4	5	6	7	8
Morning	1		117	3.1	9.6	76.1	55.5	112
Afternoon	0.8	1.5	357	5.5		23.4	321	41.3
Evening	1.3	1.1	96	6.1	3.4	120	10	22.5

Case 2 – Intervention

	1	2	3	4	5	6	7	8	9	10
Morning	102	24	4.4	18	1.1		1.3	2.2	2.5	1.9
Afternoon	23	4.2	4.7	35	0.8		2.9	4.3	2.2	0.9
Evening	38	4	2.6	5.4	7.6		2.4	3.4	4.3	167

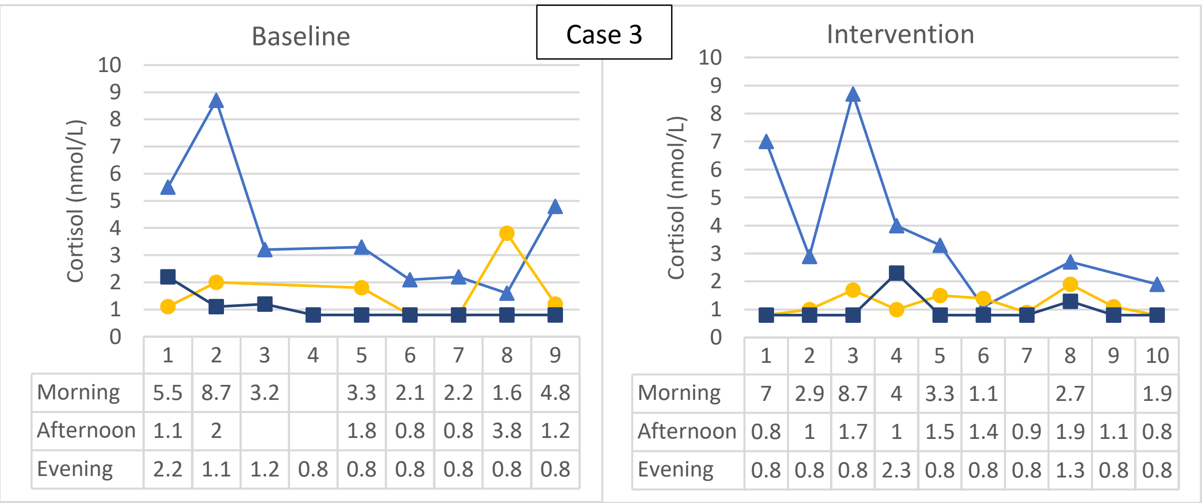

Case 3 – Baseline

	1	2	3	4	5	6	7	8	9
Morning	5.5	8.7	3.2		3.3	2.1	2.2	1.6	4.8
Afternoon	1.1	2			1.8	0.8	0.8	3.8	1.2
Evening	2.2	1.1	1.2	0.8	0.8	0.8	0.8	0.8	0.8

Case 3 – Intervention

	1	2	3	4	5	6	7	8	9	10
Morning	7	2.9	8.7	4	3.3	1.1		2.7		1.9
Afternoon	0.8	1	1.7	1	1.5	1.4	0.9	1.9	1.1	0.8
Evening	0.8	0.8	0.8	2.3	0.8	0.8	0.8	1.3	0.8	0.8

Note: x-axis = measurement occasions; y-axis=cortisol concentration in nmol/L. Imputed values used for the purposes of calculating AUC indicators have not been included. Therefore, blank cells below the graphs indicate missing samples due to insufficient saliva, or the sample not being collected by the parent on that day. Due to the large variability in Case 2, the scale on the y-axis was not standardised with Cases 1 and 3, in order to suitably visualise the raw data. Lines with triangular markers denote morning concentrations (blue); lines with circular markers denote afternoon concentrations (yellow); lines with square markers denote evening concentrations (black). The two post-intervention data points have also been omitted for ease of visualisation of baseline-to-intervention effect.
