# Supplementary material for: Using Attachment and Biobehavioral Catch-up with young children with developmental delays: A multiple-baseline trial of attachment, sensitivity, and cortisol
Source: J Intellect Disabil. 2022 Jun 1;27(4):847–70. doi: 10.1177/17446295221104614 (PMC10647892; doi:10.1177/17446295221104614)
Supplement: Supplemental Material - Using attachment and biobehavioral catch-up with young children with developmental delays: A multiple-baseline trial of attachment, sensitivity, and cortisol [file sj-pdf-2-jld-10.1177_17446295221104614.pdf]

Supplementary Table S1

Indicators of treatment fidelity for all cases.

	Case 1[a]	Case 2[b]	Case 3[c]
	**M (SD)**	**M (SD)**	**M (SD)**
Comments per minute.	1.9 (.64)	1.03 (.43)	1.08 (.45)
Average number of components.	2.04 (.29)	1.24 (.28)	1.6 (.45)
Percentage on-target.	92.04 (11.88)	91.25 (16.1)	95.49 (9.14)

[a]n = 10 (100%)
[b]n = 6 (60%)
[c]n = 8 (80%)

Supplementary Table S2

Mean level and level change for maternal sensitivity, attachment security and cortisol indicators

	**Phase Mean (SD)**			**Mean Change**		
	**A1**	**B**	**A2**	**A1/B**	**B/A2**	**A1/A2**
Sensitivity						
Case 1	4.8 (.33)	5.75 (.86)	6 (.71)	.95	.25	1.2
Case 2	6.46 (.57)	5.38 (1.73)	7[a]	-1.09	1.63	.54
Case 3	6.78 (1.06)	7.42 (.72)	7.75 (.35)	.64	.33	.97
Security						
Case 1	-.17 (0.14)	.14 (.21)	.28 (.36)	.3	.14	.45
Case 2	-.001 (.15)	.03 (.04)	.02[a]	.03	-.01	.02
Case 3	.32 (.21)	.39 (.23)	.37 (.09)	.07	-.02	.05
Cortisol Magnitude (AUC_G)						
Case 1	4.19 (1.02)	4.85 (1.2)	5.13 (.67)	.66	.28	.94
Case 2	29.86 (51.42)	36.1 (40.33)	9.78 (1.87)	6.24	-26.32	-20.08
Case 3	4.21 (1.52)	3.39 (1.47)	5.28 (4.35)	-.82	1.89	1.07
Cortisol Change (AUC_I)						
Case 1	-2.05 (4.09)	-5.46 (3.38)	-.38 (1.17)	-3.41	5.08	1.68
Case 2	-.64 (4.17)	3.14 (3.82)	-3.83 (3.22)	3.78	-6.97	-3.19
Case 3	-3.64 (3.68)	-3.33 (3.85)	-.2[a]	.31	3.13	3.44

Note: A1 = baseline phase; B = intervention phase; A2 = post-intervention phase.
[a]Raw score due to only one available data point.

Supplementary Table S3

Exploratory Theil-Sen Trend estimations for sensitivity, security and cortisol indicators

	**Theil-Sen**	**SE**	**Z**	***P***	**90% CI**
Sensitivity					
Case 1					
A1	0.04	0.17	0.24	0.81	-0.24 / 0.32
B	0.16	0.19	0.87	0.39	-0.15 / 0.48
A2	-1	1	-1	0.32	-2.65 / 0.65
Case 2					
A1	0	0	0.15	0.88	0.00 / 0.00
B	-0.1	0.4	-0.25	0.8	-0.75 / 0.57
A2[a]	-	-	-	-	-
Case 3					
A1	-0.25	0.17	-1.46	0.14	-0.53 / 0.03
B	0.25	0.13	1.88	0.06	0.03 / 0.47
A2	-0.5	0.5	-1	0.32	-1.32 / 0.32
Security					
Case 1					
A1	0.06	0.06	0.99	0.33	-0.04 / 0.15
B	0.07	0.04	1.98	0.05	0.01 / 0.13
A2	0.51	0.51	1	0.32	-0.33 / 1.34
Case 2					
A1	-0.002	0.02	-0.15	0.88	-0.03 / 0.02
B	-0.01	0.01	-1.48	0.14	-0.02 / 0.001
A2[a]	-	-	-	-	-
Case 3					
A1	-0.05	0.03	-1.46	0.14	-0.1 / 0.006
B	-0.01	0.03	-0.42	0.68	-0.06 / 0.04
A2	0.13	0.13	1	0.32	-0.08 / 0.33
Cortisol Magnitude (AUC_G)					
Case 1					
A1	-0.09	0.18	-0.49	0.62	-0.38 / 0.21
B	-0.06	0.14	-0.42	0.68	-0.28 / 0.17
A2	-0.95	0.95	-1	0.32	-2.51 / 0.61
Case 2					
A1	6.2	2.53	2.45	0.01	2.04 / 10.36
B	-2.62	1.34	-1.95	0.05	-4.83 / -0.41
A2	-2.65	2.65	-1	0.32	-7.01 / 1.71
Case 3					
A1	-0.37	0.38	-0.99	0.32	-0.99 / 0.25
B	-0.29	0.14	-2.06	0.04	-0.52 / -0.06
A2	-6.15	6.15	-1	0.32	-16.27 / 3.97
Cortisol Change (AUC_I)					
Case 1					
A1	1.66	1.7	0.98	0.33	-1.13 / 4.45
B	-0.54	0.46	-1.16	0.24	-1.3 / 0.22
A2	1.65	1.65	1	0.32	-1.06 / 4.36
Case 2					
A1	-1.1	1.62	-0.68	0.5	-3.76 / 1.56
B	-0.4	0.44	-0.9	0.37	-1.13 / 0.33
A2	4.55	4.55	1	0.32	-2.94 / 12.04
Case 3					
A1	0.81	0.65	1.24	0.22	-0.27 / 1.88
B	0.74	0.44	1.7	0.09	0.02 / 1.46
A2[a]	-	-	-	-	-

Note: $p \leq .05$

[a]Only one data point available
